# Impact of rituximab-based desensitization on T cell-mediated rejection in ABO-incompatible liver transplantation

**DOI:** 10.1007/s00595-025-03162-3

**Published:** 2026-01-05

**Authors:** Kengo Sasaki, Kazuaki Tokodai, Atsushi Fujio, Muneyuki Matsumura, Yoshihiro Shono, Hiroyuki Ogasawara, Ryusuke Saito, Naruhito Takido, Michiaki Unno, Takashi Kamei

**Affiliations:** Department of Surgery, Tohoku University Graduate School of Medicine, 1-1 Seiryo-machi, Aoba-ku, Sendai, 980-8575 Japan

**Keywords:** Liver transplantation, T cell-mediated rejection, Rituximab, Acute rejection, Acute cellular rejection

## Abstract

**Purpose:**

Rituximab-based desensitization has enabled successful ABO-incompatible (ABO-I) liver transplantation (LT) by preventing antibody-mediated rejection (AMR). However, its effect on T cell-mediated rejection (TCMR) remains unclear. We conducted a comparative analysis between ABO-compatible (ABO-C) and ABO-I LT to evaluate the effects of rituximab-based desensitization on TCMR.

**Methods:**

We retrospectively analyzed 45 LT recipients (32 ABO-C and 13 ABO-I recipients) treated with basiliximab-based immunosuppression. The ABO-I group additionally received rituximab-based desensitization therapy. The lymphocyte subpopulations, rejection, adverse events, and outcomes were assessed.

**Results:**

AMR was not observed in either group. TCMR occurred within 4 weeks post-transplantation in 0% of ABO-C cases and 38.5% of ABO-I cases (*P* = 0.0011). In ABO-C, a significant increase in B cells (CD19+) was observed within the first week, whereas in ABO-I, B cells remained depleted and an increase in T cells (CD3+) was observed. In all the ABO-I cases, TCMR occurred under suppressed CD25 + conditions. Adverse events were comparable between the groups. The 1-year survival rates for the ABO-C and ABO-I groups were 96.9% and 100%, respectively.

**Conclusion:**

Rituximab-based desensitization in ABO-I LT is associated with an increased incidence of early TCMR. Rituximab-induced B-cell depletion may promote T-cell activation through an IL-2-independent pathway, potentially contributing to increased TCMR.

## Introduction

Over the past few decades, the outcomes of liver transplantation (LT) have significantly improved, establishing it as the standard treatment for end-stage liver disease. These improvements are largely attributable to advances in immunosuppressive therapy, donor selection, surgical techniques, and perioperative management [1–7]. Biological immunosuppressive agents, including polyclonal and monoclonal antibodies, target surface molecules on immune cells, and are used in induction therapy, desensitization therapy for ABO-incompatible (ABO-I) transplants, and steroid-resistant rejection [1, 8–10]. Additionally, these agents help to reduce the use of calcineurin inhibitors and steroids, thereby minimizing immunosuppressant-related side effects [11, 12].

Basiliximab is a chimeric monoclonal antibody that specifically targets CD25 and the alpha chain of interleukin (IL)−2 receptor. By binding to the IL-2 receptor, which is predominantly expressed on activated T cells, basiliximab inhibits T-cell proliferation. Basiliximab induction therapy has been reported to enhance graft survival with fewer side effects and early acute rejections [1, 13–15].

Rituximab is a chimeric monoclonal antibody that targets CD20 and is expressed in most B cells. Rituximab-based desensitization therapy for ABO-I LT has significantly reduced the incidence of antibody-mediated rejection (AMR) and improved outcomes, successfully expanding the donor pool [8, 9, 16]. However, despite reducing AMR, B-cell depletion by rituximab has also been reported to increase the incidence of acute cellular rejection (ACR) after kidney transplantation [17]. To date, no detailed studies have examined the effect of rituximab on T-cell-mediated rejection (TCMR) in LT.

In this study, we conducted a comparative analysis of ABO-compatible (ABO-C) and ABO-I LT to investigate the effects of rituximab-induced B-cell depletion on the occurrence of TCMR.

## Methods

### Study design

**Fig. 1:**
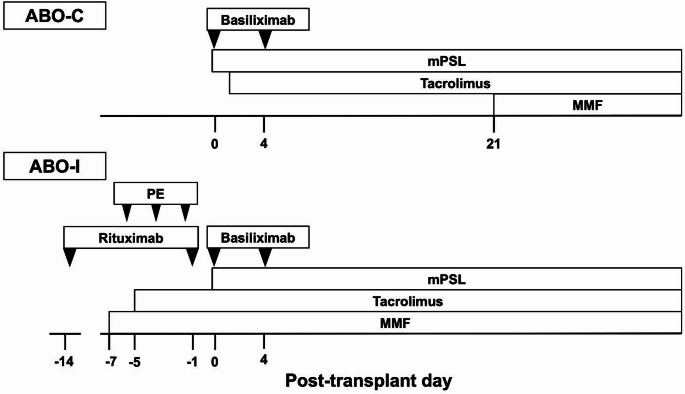
Immunosuppressive regimens for ABO-C and ABO-I. ABO-C, ABO-compatible; ABO-I, ABO-incompatible; MMF, mycophenolate mofetil; mPSL, methylprednisolone; PE, plasma exchange

Our current immunosuppressive regimens include basiliximab, methylprednisolone, tacrolimus, and mycophenolate mofetil (MMF) for ABO-C LT and rituximab for ABO-I LT (Fig. 1) [18]. This study included patients who underwent LT at Tohoku University Hospital under these immunosuppressive regimens until March 2024. We collected clinical data of recipient characteristics, including sex, age, weight, height, body mass index (BMI), primary disease, model for end-stage liver disease (MELD) score, donor type, flow cytometry cross-match (FCXM), renal impairment, operative time, intraoperative blood loss, cold ischemia time (CIT), splenectomy, and graft-to-recipient weight ratio (GRWR), and then collected and assessed post-transplant data on lymphocyte subpopulations, rejection, adverse events and outcomes, comparing cases of ABO-C and ABO-I LT.

This study was approved by the Ethics Committee of Tohoku University (approval number: 2022-1-945). The requirement for informed consent was waived owing to the retrospective nature of the study. Instead, information about the study was made publicly available on the institutional website, allowing patients the opportunity to opt out.

## Immunosuppressive regimen for ABO-C transplantation

In ABO-C LT, immunosuppressive therapy was initiated on the day of the transplantation. Methylprednisolone was administered intravenously at a dose of 1,000 mg during surgery, tapered from 200 mg to 20 mg on postoperative day (POD) 14, switched to an oral dose of 16 mg, and gradually tapered off by 3 months after surgery. Basiliximab was administered intravenously at a dose of 20 mg/body, the first dose during surgery and the second on POD 4. Tacrolimus was administered at a target trough level of 8–10 ng/mL from POD 1 to 27 and 6–8 ng/mL from POD 28 to 84. MMF was initiated at a dose of 1,000 mg/day on POD 21.

At our institution, no additional pretransplant intervention was undertaken for FCXM-positive recipients beyond the standard ABO-C regimen, and this policy was also applied to the FCXM-positive cases included in this study.

## Immunosuppressive regimen for ABO-I transplantation

Preoperative desensitization therapy for ABO-I LT included rituximab, MMF, tacrolimus, and plasma exchange. Rituximab was administered at 300 mg/body 2 weeks before LT and at 200 mg/body the day before LT. MMF was initiated 7 days before LT at a dose of 1,500 mg/day, and tacrolimus was initiated 5 days before LT at a target trough level of 5–8 ng/mL. Plasma exchange was performed 1–3 times before LT, depending on the anti-ABO antibody titer and the patient’s general condition before surgery. Intraoperative and postoperative administration of methylprednisolone, basiliximab, and tacrolimus were performed in the same manner as in ABO-C LT. MMF was continued at the same preoperative dose of 1,500 mg/day after LT.

## Graft selection

In our graft selection criteria for living-donor liver transplantation (LDLT), the estimated graft volume-to-standard liver volume ratio was required to be ≥ 40%, as assessed using volumetric imaging. A left lobe graft was prioritized when this threshold was met to preserve a greater remnant liver volume for the donor. The donor remnant liver volume was required to be at least 30% for donors younger than 41 years of age and at least 35% for those of 41 years of age or older. For deceased-donor liver transplantation, grafts were allocated in accordance with the Japan Organ Transplant Network policy, with suitability confirmed by the transplant team based on the donor quality and recipient condition.

## Flow cytometry analysis

The lymphocyte subpopulations were analyzed using flow cytometry. Data were collected on the proportions of CD3+, CD3 + CD4+, CD3 + CD8+, CD3 + CD25+, and CD19 + lymphocytes at the following time points: at the time of transplantation (on the day of or 24 h before surgery), and postoperative week (POW) 1 (POD 6–8), week 2 (POD 13–15), week 3 (POD 20–22), week 4 (POD 27–29), and week 5 (POD 34–36). As rituximab induces profound B-cell depletion, the relative lymphocyte proportions can be distorted and may not be suitable for cross-group comparisons. Therefore, temporal changes in proportions were analyzed within each group by comparing adjacent time points, whereas between-group comparisons were performed using absolute counts.

### Rejection

Rejection was histologically diagnosed through a liver biopsy according to the Banff criteria [19]. The rejection activity index and C4d staining by immunohistochemistry (immunoperoxidase method) were evaluated according to Banff criteria. C4d deposition was scored as follows: 0 (negative), 1 (minimal), 2 (focal), or 3 (diffuse). TCMR treatment followed a standardized protocol: high-dose methylprednisolone pulse was used as first-line therapy, and anti-thymocyte globulin was administered as second-line treatment for steroid-refractory cases. The incidence of rejection was compared between the ABO-C and ABO-I groups at 4-week intervals during the first 12 weeks after LT. The cumulative rejection-free rate within the first year after transplantation was analyzed using Kaplan–Meier curves and compared between the ABO-C and ABO-I groups.

## Adverse events and outcomes

We collected data on infectious complications within three months after LT, renal impairment at three months after LT, surgical complications, post-transplant lymphoproliferative disorders (PTLD), and mortality within one year after LT. Infectious complications included cytomegalovirus (CMV) infection, CMV disease, other viral infections, organ/space surgical site infection (SSI), and bacteremia. As a strategy against CMV infection, we adopted preemptive rather than prophylactic therapy while monitoring CMV using pp65 antigenemia or a quantitative real-time polymerase chain reaction assay. CMV infection was defined as cases in which the patients received preemptive therapy. CMV disease was defined as the presence of an organ disorder, such as pneumonia, gastrointestinal disease, or hepatitis, and was confirmed by histological findings specific to CMV. Organ/space SSI was defined as the presence of signs of infection with a positive ascitic fluid culture or imaging findings indicating an intra-abdominal infectious focus. Bacteremia was defined as the presence of signs of infection with a positive blood culture. Renal impairment was defined as an estimated glomerular filtration rate (eGFR) of less than 40 mL/min/1.73 m². Transplant outcomes within 1 year were evaluated based on surgical complications including hepatic or portal vein stenosis, hepatic artery thrombosis (HAT), biliary stricture or bile leakage, PTLD, mortality, or death associated with infection.

### Statistical analysis

All statistical analyses were performed using JMP Pro (ver. 17.1.0, SAS Institute Inc., Cary, NC, USA). Qualitative data were expressed as frequencies and percentages and analyzed using Fisher’s exact test. Quantitative data were expressed as the mean and standard deviation and analyzed using Student’s t-test for parametric data and the Mann–Whitney test for nonparametric data. The cumulative rejection-free rate was analyzed using Kaplan–Meier curves, and differences were analyzed using the log-rank test. Two-sided P-values of < 0.05 were considered to indicate statistical significance. To identify independent risk factors for TCMR, we performed a multivariable logistic regression analysis using Firth’s penalized likelihood method to account for the small sample size and sparse outcomes. The variables included in the model were ABO compatibility, recipient age, and CIT. Sensitivity analyses were also conducted by incorporating splenectomy status and restricting the cohort to LDLT recipients.

## Results

### Recipient characteristics

Under our current immunosuppressive regimen, 45 patients underwent LT, including 32 patients with ABO-C and 13 with ABO-I. The recipient characteristics are summarized in Table 1. No significant differences were observed in terms of patient sex, age, weight, BMI, primary disease, MELD score, FCXM, intraoperative blood loss, GRWR, or rate of cases with eGFR < 40 mL/min/1.73 m². Significant differences were observed in terms of height, donor type, operative time, CIT, and splenectomy status. All ABO-I cases were living-donor liver transplantations, a factor that significantly differed from ABO-C cases, and likely contributed to the observed differences in operative time and CIT. Splenectomy was performed more frequently in the ABO-I group than in the ABO-C group.

**Table 1:** Recipient characteristics

	ABO-C	ABO-I	*P* value
	*n* = 32	*n* = 13	
Sex, male	19 (59.4%)	7(53.8%)	0.75
Age, years	44.5±11.6	39.7±19.2	0.35
Weight, kg	63.7±12.1	57.1±14.8	0.15
Height, cm	166.6±7.7	156.0±14.3	0.0041**
BMI, kg/m^2^	22.9±3.7	23.2±4.8	0.84
Primary disease			0.25
Biliary atresia	4 (12.5%)	4 (30.8%)	
Primary sclerosing cholangitis	4 (12.5%)	0	
Primary biliary cholangitis	4 (12.5%)	1 (7.7%)	
Acute liver failure	5 (15.6%)	0	
Liver cirrhosis			
HCV	1 (3.1%)	0	
HBV	0	1 (7.7%)	
Alcoholic	5 (15.6%)	1 (7.7%)	
MASH	1 (3.1%)	1 (7.7%)	
Cryptogenic	1 (3.1%)	1 (7.7%)	
HCC	4 (12.5%)	2 (15.4%)	
Wilson’s disease	0	1 (7.7%)	
Budd Chiari	2 (6.3%)	0	
Others	1 (3.1%)	1 (7.7%)	
MELD score	22.1±10.5	17.7±7.2	0.18
Donor type			0.0087**
Living-donor	19 (59.4%)	13 (100%)	
Deceased donor	13 (40.6%)	0	
FCXM			
T cell (+)	7 (21.9%)	4 (30.8%)	0.7
B cell (+)	7 (21.9%)	4 (30.8%)	0.7
eGFR < 40 mL/min/1.73 m^2^	4 (12.5%)	2 (15.4%)	1
Operative time, min	791.7±219.5	959.8±191.2	0.020*
Intraoperative blood loss, mL	9,086.4±10,671.2	7,542.2±4,841.4	0.62
Cold ischemia time, min	307.9±195.4	157.9±76.1	0.011*
Splenectomy	12 (37.5%)	10 (76.9%)	0.023*
GRWR, %	1.44±0.72	1.25±0.67	0.42

ABO-C, ABO-compatible; ABO-I, ABO-incompatible; BMI, body mass index; eGFR, estimated glomerular filtration rate; FCXM, flow cytometry cross-match; GRWR, graft-to-recipient weight ratio; HBV, hepatitis B virus; HCC, hepatocellular carcinoma; HCV, hepatitis C virus; MASH, metabolic dysfunction associated with steatohepatitis; MELD, model of end-stage liver disease; *, *P* < 0.05; **, *P* < 0.01

### Lymphocyte subpopulations

**Fig. 2:**
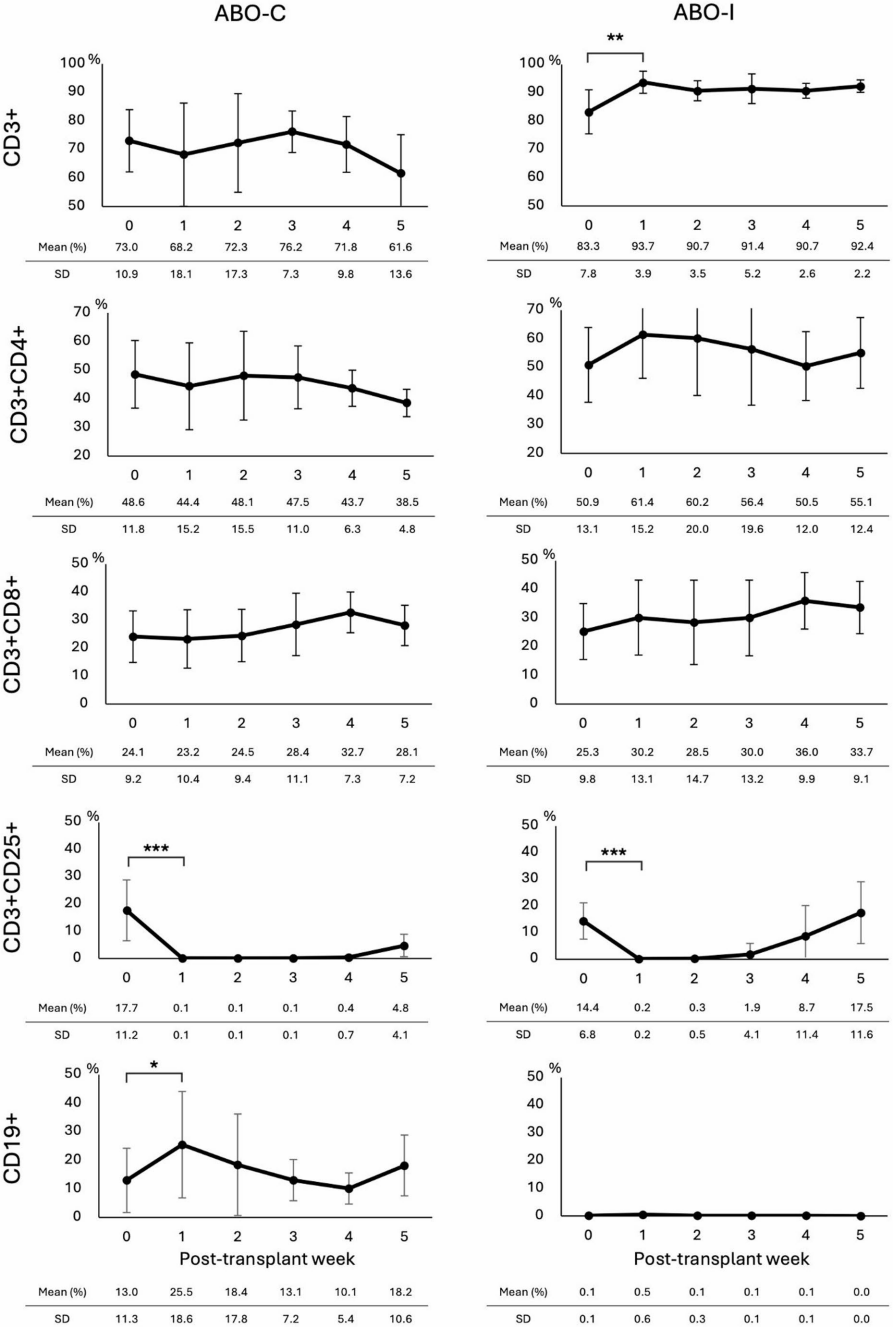
Proportions of lymphocyte subpopulations. ABO-C, ABO-compatible; ABO-I, ABO-incompatible; SD, standard deviation; *, *P* < 0.05; **, *P* < 0.01; ***, *P* < 0.0001.

The proportions of the lymphocyte subpopulations are shown in Fig. 2. The proportion of CD3 + lymphocytes, specifically T cells, did not significantly change in the ABO-C group, whereas a significant increase was observed in the ABO-I group within the first week after transplantation (*P* = 0.0021). No significant changes were observed in the proportions of CD3 + CD4 + or CD3 + CD8 + lymphocytes in either group. The proportion of CD3 + CD25 + lymphocytes significantly decreased in both groups within the first week post-transplantation (*P* < 0.0001 in both groups) and remained markedly suppressed during the first three weeks after transplantation. The proportion of CD19 + lymphocytes (specifically B cells) significantly increased within the first week post-transplantation in the ABO-C group (*P* = 0.035), while it remained less than 1% from pre-transplantation through postoperative week 5 in the ABO-I group. The absolute count data of lymphocyte subpopulations are shown in Supplementary Fig. 1. CD19 + cells showed significant differences at all time points, whereas the absolute counts of CD3+, CD4+, CD8+, and CD25 + cells did not differ significantly between the groups.

### Rejection

**Fig. 3:**
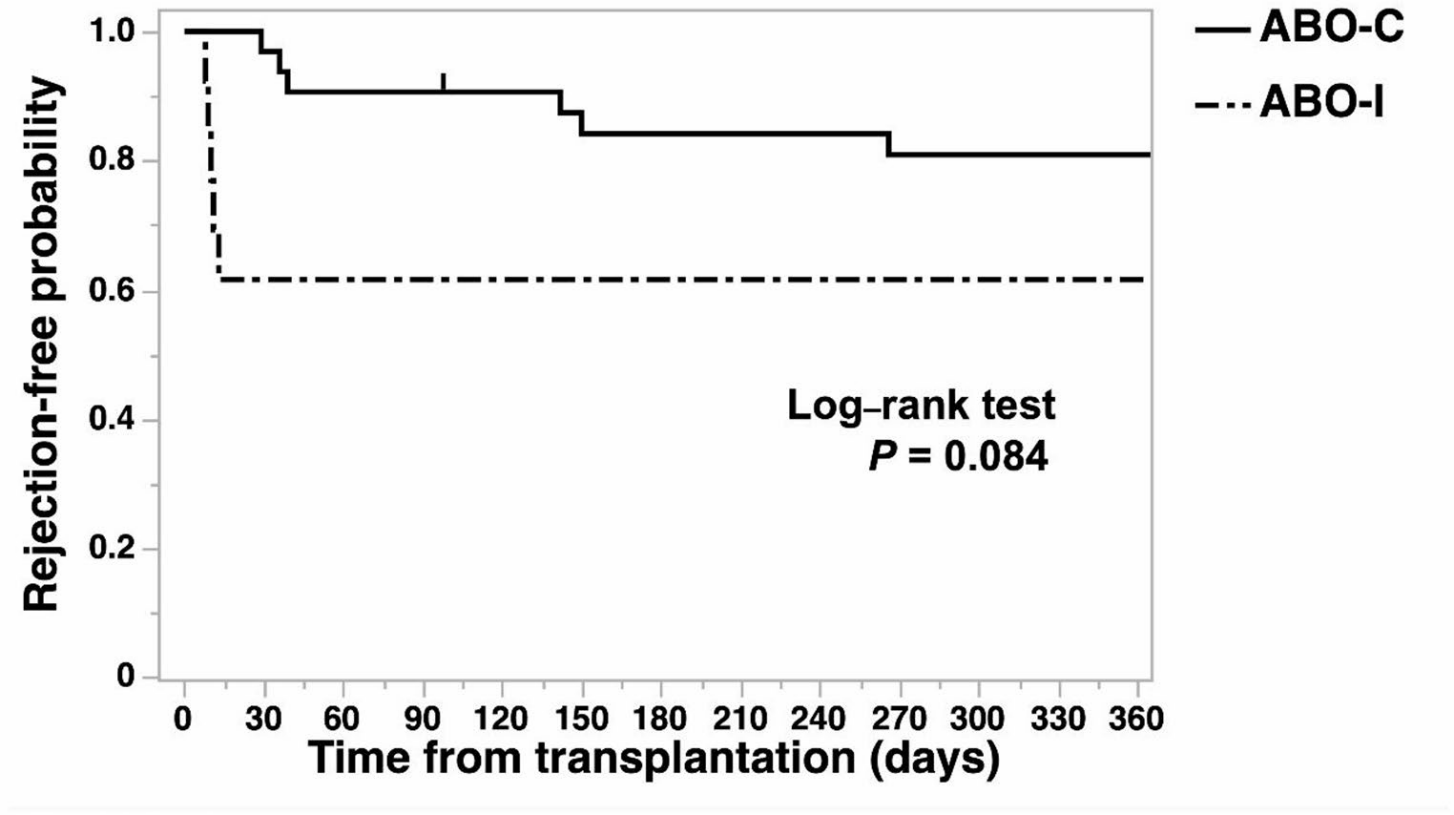
Kaplan–Meier curves for rejection–free probability. ABO-C, ABO-compatible; ABO-I, ABO-incompatible

Within the first year after transplantation, no definite AMR was observed in either ABO-C or ABO-I. TCMR was observed in 9.4% of ABO-C cases and 38.5% of ABO-I cases within 12 weeks post-transplantation, with a significant difference (*P* = 0.034) (Table 2). The immunological and histopathological characteristics of the rejection cases are shown in Table 3. Although three ABO-I cases demonstrated C4d staining, C4d deposition was minimal (score 1), and histological findings fulfilled the Banff criteria for TCMR, all patients were diagnosed with TCMR rather than AMR. The incidence of TCMR at each 4-week interval was as follows: in ABO-C, 0% in POW 1–4, 9.4% in POW 5–8, and 0% in POW 9–12; and in ABO-I, 38.5% in POW 1–4, 0% in POW 5–8, and 0% in POW 9–12. A significant difference was found in the frequency of TCMR during POW 1–4 (*P* = 0.0011). The Kaplan–Meier curves for the probability of rejection-free survival are shown in Fig. 3. The log-rank test did not reveal a statistically significant difference. In the multivariate logistic regression analysis (Tables 4, 5, 6, 7 and 8), ABO incompatibility was not identified as an independent risk factor for overall TCMR within 12 weeks. However, when focusing on TCMR occurring within four weeks, ABO-I showed a strong trend toward increased risk. Sensitivity analyses adjusted for splenectomy, with the analysis restricted to LDLT cases, yielded consistent results, with ABO-I being associated with higher odds of early TCMR, although this was not statistically significant.

**Table 2:** Incidence and timing of T cell-mediated rejection

Post-transplant period	ABO-C	ABO-I	*P* value
	*n*=32	*n*=13	
POW 1–4	0	5 (38.5%)	0.0011**
POD 1–7	0	0	
POD 8–14	0	5 (38.5%)	0.0011**
POD 15–21	0	0	
POD 22–28	0	0	
POW 5–8	3 (9.4%)	0	0.55
POW 9–12	0	0	
Total	3 (9.4%)	5 (38.5%)	0.034*

ABO-C, ABO-compatible; ABO-I, ABO-incompatible; POD, postoperative day; POW, postoperative week; *, *P* < 0.05; **, *P* < 0.01

**Table 3:** Immunological and histopathological characteristics of rejection cases

Case	ABO compatibility	FCXM		Anti-donor ABO Ab titer		Histopathology				Anti-donor ABO Ab titer		DSA at Dx	Treatment for rejection
		before Tx		at Tx						at Dx			
		T cell	B cell	IgM	IgG	Dx	RAI	C4d score	Date of Dx (POD)	IgM	IgG		
1	Compatible	-	-			TCMR	4	0	29			N/A	Steroid pulse
2	Compatible	-	-			TCMR	4	0	36			N/A	Steroid pulse
3	Compatible	-	-			TCMR	5	0	39			-	Steroid pulse
4	Incompatible	-	-	16	<2	TCMR	7	0	8	1	<2	N/A	Steroid pulse
5	Incompatible	-	-	16	<2	TCMR	4	0	11	1	<2	N/A	Steroid pulse
6	Incompatible	+	+	64	<2	TCMR	6	1	9	<1	<2	N/A	Steroid pulse ATG
7	Incompatible	-	-	32	4	TCMR	5	1	13	2	<2	N/A	Steroid pulse
8	Incompatible	-	-	16	16	TCMR	5	1	10	1	4	N/A	Steroid pulse

Ab, antibody; ATG, anti-thymocyte globulin; DSA, donor-specific antibody; Dx, diagnosis; FCXM, flow cytometry cross-match; N/A, not applicable; POD, postoperative day; RAI, rejection activity index; TCMR, T cell-mediated rejection; Tx, transplantation

**Table 4:** Multivariable analysis for TCMR ≤12 weeks

Variable	aOR	95% CI	*P* value
ABO-I (vs. ABO-C)	2.62	0.41–16.78	0.31
Age (per 10 years)	0.59	0.35–1.01	0.053
CIT (per 60 min)	0.95	0.67–1.36	0.79

**Table 5:** Multivariable analysis for TCMR ≤4 weeks

Variable	aOR	95% CI	*P* value
ABO-I (vs. ABO-C)	13.73	0.93–203.73	0.057
Age (per 10 years)	0.66	0.36–1.21	0.18
CIT (per 60 min)	0.79	0.37–0.17	0.55

**Table 6:** Sensitivity analysis including splenectomy

Variable	aOR	95% CI	*P* value
ABO-I (vs. ABO-C)	2.04	0.37–14.34	0.37
Age (per 10 years)	0.58	0.33–0.99	0.047
CIT (per 60 min)	0.99	0.67–1.48	0.97
Splenectomy (yes vs. no)	1.75	0.24–12.59	0.58

**Table 7:** LDLT-only multivariable analysis for TCMR ≤12 weeks

Variable	aOR	95% CI	*P* value
ABO-I (vs. ABO-C)	3.06	0.46–20.21	0.25
Age (per 10 years)	0.66	0.39–1.14	0.14
CIT (per 60 min)	0.71	0.28–1.79	0.47

**Table 8:** LDLT-only multivariable analysis for TCMR ≤4 weeks

Variable	aOR	95% CI	*P* value
ABO-I (vs. ABO-C)	16.81	0.86–327.52	0.063
Age (per 10 years)	0.68	0.36–1.27	0.23
CIT (per 60 min)	0.68	0.23–2.03	0.48

ABO-C, ABO-compatible; ABO-I, ABO-incompatible; aOR, adjusted odds ratio; CI, confidence interval; CIT, cold ischemia time; LDLT, living-donor liver transplantation; TCMR, T cell-mediated rejection

In the ABO-C group, no TCMR was observed within the first 4 weeks, and TCMR occurred only after the proportion of CD3 + CD25 + lymphocytes began to increase beyond 4 weeks. In ABO-I, all TCMR cases were observed within the first two weeks, with the proportion of CD3 + CD25 + lymphocytes at the time of rejection ranging from 0.0% to 0.7%.

### Adverse events and outcomes

The adverse events and outcomes are summarized in Table 9. CMV infection was observed in 43.8% of ABO-C cases and 53.9% of ABO-I cases; however, CMV disease did not occur in either group. Other viral infections were identified in 6.3% and 7.7% of ABO-C and ABO-I cases, respectively. Organ/space SSI occurred in 31.3% of ABO-C cases and 46.2% of ABO-I cases, whereas bacteremia occurred in 6.3% and 15.4% of ABO-C and ABO-I cases, respectively. No significant differences in any of these infectious complications were observed between the two groups.

**Table 9:** Adverse events and outcomes

Adverse events	ABO-C	ABO-I	*P* value
	*n*=32	*n*=13	
Within 3 months			
CMV infection	14 (43.8%)	7 (53.9%)	0.74
CMV disease	0	0	
Other viral infection	2 (6.3%)	1 (7.7%)	1
Organ/space SSI	10 (31.3%)	6 (46.2%)	0.49
Bacteremia	2 (6.3%)	2 (15.4%)	0.57
At 3 months			
eGFR < 40 mL/min/1.73 m^2^	1 (3.1%)	0	1
Within 12 months			
Hepatic vein stenosis	1 (3.1%)	0	1
Portal vein stenosis	0	1 (7.7%)	0.29
Hepatic artery thrombosis	1 (3.1%)	0	1
Biliary stricture	4 (12.5%)	1 (7.7%)	1
Bile leakage	7 (21.9%)	2 (15.4%)	1
PTLD	0	0	
Mortality	1 (3.1%)	0	1
Death associated with infection	1 (3.1%)	0	1
1-year survival rate	96.90%	100%	

ABO-C, ABO-compatible; ABO-I, ABO-incompatible; CMV, cytomegalovirus; eGFR, estimated glomerular filtration rate; PTLD, post-transplant lymphoproliferative disorder; SSI, surgical site infection

Although cases with an eGFR < 40 mL/min/1.73 m² accounted for 12.5% of ABO-C cases and 15.4% of ABO-I cases before LT, these rates decreased to 3.1% and 0%, respectively, at 3 months post-transplantation.

No significant differences in surgical complications were observed between the two groups. PTLD did not occur in any of the groups. Within the first year, only one death was observed in the ABO-C group; the death was associated with infection. The incidence of death did not differ significantly between the groups.

## Discussion

This study compared ABO-C and ABO-I LT to evaluate the efficacy and safety of our immunosuppressive regimens and investigate the occurrence of TCMR. These findings revealed differences in the incidence and timing of TCMR. A more intensive immunosuppressive regimen was employed for ABO-I than for ABO-C, including preoperative rituximab administration and the early initiation of tacrolimus and MMF; therefore, a lower incidence of TCMR was anticipated. Although this regimen effectively suppressed AMR, the incidence of TCMR during the first 12 weeks after transplantation, particularly within the first 4 weeks, was significantly higher in the ABO-I group than that in the ABO-C group. The multivariable analysis did not demonstrate ABO incompatibility as an independent predictor of overall TCMR within 12 weeks but suggested a trend toward an increased risk of early TCMR within 4 weeks, supporting our observation that rejection was clustered in the early post-transplant period.

The addition of rituximab may have contributed to an increased incidence of early TCMR. Clatworthy et al. reported that B cell-depleting therapy with rituximab increased the risk of ACR within the first three months after kidney transplantation [17]. They suggested that depletion of regulatory B cells (Bregs) may have contributed to increased rejection in patients treated with rituximab. Bregs are a specialized subset of B cells, characterized by their ability to suppress immune responses and maintain immune homeostasis. Bregs exert their effects mainly through the secretion of anti-inflammatory cytokines such as IL-10, IL-35, and transforming growth factor-beta [20]. Bregs have been implicated in the pathogenesis of allergies and autoimmune diseases [21, 22]. Furthermore, B cell-depleting therapy with rituximab has been associated with colitis and exacerbation of autoimmune diseases, suggesting that the suppression of Bregs by rituximab may contribute to the activation of immune responses and disease exacerbation [23, 24]. In the field of organ transplantation, several studies have reported the involvement of Bregs in immune tolerance after kidney transplantation [25–29]. However, few studies have investigated the activation of the immune system and the increased incidence of rejection caused by the suppression of Bregs. Our study is the first to demonstrate that the same phenomenon reported by Clatworthy et al. occurs in LT patients.

Importantly, our data revealed distinct post-transplant lymphocyte dynamics between ABO-C and ABO-I, which corresponded to the timing of TCMR. In the ABO-C group, the proportion of CD19 + lymphocytes significantly increased during the first week after LT. During this period, CD25 + T cells were markedly suppressed and no episodes of TCMR occurred. TCMR in ABO-C emerged only after four weeks, coinciding with the recovery of CD25 + T cells, suggesting that rejection in this group followed an IL-2-dependent pathway of T cell activation. In contrast, rituximab prevented the early post-transplant increase in CD19 + lymphocytes in the ABO-I group. However, a significant increase in the proportion of CD3 + lymphocytes was observed within the first week. Strikingly, all TCMR episodes in ABO-I occurred during this early period when CD25 + T cells were strongly suppressed. These findings suggest that the absence of an early post-transplant increase in CD19 + lymphocytes due to rituximab-induced B-cell depletion is likely a key factor contributing to the increased incidence of early TCMR. This lack of B cell recovery may also have facilitated the observed increase in CD3 + lymphocytes and subsequent T cell activation, although this association remains unclear. Moreover, the fact that TCMR occurs during a period of profound CD25 suppression raises the possibility that T cell activation proceeds through an IL-2-independent pathway.

Tanaka et al. reported experimental evidence demonstrating that rituximab-induced B-cell depletion paradoxically enhances donor-reactive CD4 + T cell responses through the loss of IL-10-producing Bregs [30]. In our study, although the increase in the proportion of CD4 + T cells did not reach statistical significance, a trend toward higher CD4 + T cell proportions was observed in the ABO-I group. This mechanism provides a plausible explanation for the increased incidence of early TCMR in ABO-I in our cohort, in which rituximab-based desensitization was uniformly applied.

Although the immunosuppressive regimen for ABO-I was more intensive, there was no significant increase in infectious complications. Despite the high incidence of CMV infection, no cases of CMV disease occurred in either group, supporting the effectiveness of the pre-emptive therapy strategy. In both groups, the proportion of patients with an eGFR < 40 mL/min/1.73 m² had improved by 3 months post-transplantation, indicating favorable renal function outcomes. Surgical complications, such as hepatic vein stenosis, portal vein stenosis, biliary stricture, or bile leakage, were infrequent and did not differ significantly between the groups. The low mortality rates within 1 year in both groups support the overall safety and feasibility of these regimens.

This is the first report suggesting that rituximab-based desensitization therapy may potentially induce TCMR in LT, and is therefore clinically significant. However, this study had some limitations. First, the small sample size, particularly for the ABO-I, limits the generalizability of the findings. Large-scale studies are required to validate our findings. Second, the retrospective design of this study may have introduced a selection bias and limited our ability to establish causality. Third, comparisons between ABO-C and ABO-I inevitably reflect differences in patient characteristics (e.g., donor type, splenectomy status, operative time, and CIT), which may have influenced the outcomes. To specifically address these potential confounders, we performed additional multivariate analyses incorporating splenectomy and restricting the cohort to LDLT recipients. These analyses yielded consistent results, showing that ABO incompatibility was associated with a higher risk of early TCMR, although this association was not statistically significant. Furthermore, desensitization procedures other than rituximab, such as plasmapheresis, may have also influenced the observed outcomes. However, in this study, we considered the influence of these factors to be minimal. Finally, because this was a single-center study, the findings may not be generalizable to institutions with different patient populations or treatment protocols. Addressing these limitations in future research will be essential to deepen our understanding of immunosuppressive and desensitization strategies for ABO-I LT.

## Conclusion

This study showed that rituximab-based desensitization for ABO-I LT is associated with an increased incidence of early TCMR, likely due to B-cell depletion and loss of Breg function. However, episodes of TCMR were manageable, and AMR, which is often more critical, was effectively controlled. Given the favorable 1-year survival rate, the ABO-I regimen remains a valuable therapeutic option. Future research should aim to elucidate the immunological mechanisms underlying these findings and optimize protocols to improve the outcomes.
